# Draft Genome Sequence of Robinsoniella peoriensis Strain WTD, Isolated from the Fecal Material of a Wood Turtle

**DOI:** 10.1128/genomeA.01444-14

**Published:** 2015-01-15

**Authors:** Jeanna L. Braasch, Carly N. Lapin, Scot E. Dowd, Richard W. McLaughlin

**Affiliations:** a General Studies, Gateway Technical College, Kenosha, Wisconsin, USA; b Wisconsin Department of Natural Resources, Rhinelander, Wisconsin, USA; c Molecular Research LP (MR DNA), Shallowater, Texas, USA; d Biology Department, Saint Mary’s University of Minnesota, Winona, Minnesota, USA

## Abstract

Here, we report the draft genome of Robinsoniella peoriensis strain WTD, which was isolated from the fecal material of a wood turtle. The genome size was 7,391,415 bp with 41.1 mol% G+C.

## GENOME ANNOUNCEMENT

Robinsoniella peoriensis was identified in 2009 as an anaerobic Gram-positive rod that is capable of forming endospores. It was originally isolated from a swine-manure storage pit (1, 2). In humans, there have been several reported cases of R. peoriensis infection (3–7). In this study, R. peoriensis strain WTD was isolated from the fecal material of a wood turtle using the ethanol shock method (8). Cells were plated on blood agar and incubated for 48 h under anaerobic conditions.

A colony of R. peoriensis was sent to Molecular Research LP (MR DNA; http://www.mrdnalab.com). DNA was isolated using the Qiagen mini DNA isolation kit (Qiagen Inc.). Afterward, using an Ion Express library kit and the Ion Torrent PGM sequencer (Life Technologies), a draft genome was sequenced following the manufacturer’s guidelines. Using NGEN (DNAstar), the genome was assembled and annotation of the genome was performed using RAST (http://rast.nmpdr.org) (9).

In total, 32 contigs with protein-encoding genes were constructed, which totaled 7,391,415 bp with a G+C content of 41.1%. Annotation in RAST revealed 73 RNAs, 6,115 coding sequences, and 370 subsystems. There were 129 genes involved in virulence, disease, and defense. Some of these genes are involved in vancomycin, fluoroquinolones, and tetracycline resistance.

The availability of this draft genome may aid in determining the role of this bacterium in human illnesses.

### Nucleotide sequence accession numbers.

This whole-genome shotgun project has been deposited at DDBJ/EMBL/GenBank under the accession number JTGN00000000. The version described in this paper is version JTGN01000000.

## References

[1] Cotta MA, Whitehead TR, Zeltwanger RL 2003 Isolation, characterization and comparison of bacteria from swine faeces and manure storage pits. Environ Microbiol 5:737–745. doi:10.1046/j.1467-2920.2003.00467.x. 12919409

[2] Cotta MA, Whitehead TR, Falsen E, Moore E, Lawson PA 2009 *Robinsoniella peoriensis* gen. nov., sp. nov., isolated from a swine-manure storage pit and a human clinical source. Int J Syst Evol Microbiol 59:150–155. doi:10.1099/ijs.0.65676-0. 19126740

[3] Shen D, Chen R, Ye L, Luo Y, Tang YW 2010 *Robinsoniella peoriensis* bacteremia in a patient with pancreatic cancer. J Clin Microbiol 48:3448–3450. doi:10.1128/JCM.00477-10. 20631102 PMC2937667

[4] López P, Belda S, García M, Royo G 2010 Infection of a spontaneous muscular hematoma due to *Robinsoniella peoriensis*, in a patient with alcoholic liver cirrhosis. Enferm Infecc Microbiol Clin 28:565–567. doi:10.1016/j.eimc.2010.01.005 (In Spanish.). 20627461

[5] Gomez E, Gustafson DR, Colgrove R, Ly T, Santana R, Rosenblatt JE, Patel R 2011 Isolation of *Robinsoniella peoriensis* from four human specimens. J Clin Microbiol 49:458–460. doi:10.1128/JCM.01305-10. 21048018 PMC3020413

[6] Ferraris L, Aires J, Butel MJ 2012 Isolation of *Robinsoniella peoriensis* from the feces of premature neonates. Anaerobe 18:172–173. doi:10.1016/j.anaerobe.2011.11.007. 22155447

[7] Cassir N, Laget L, Renvoisé A, Gennari JM, Drancourt M 2012 *Robinsoniella peoriensis* infection following surgery for scoliosis: a case report. J Med Case Rep 6:174. doi:10.1186/1752-1947-6-174. 22742769 PMC3407743

[8] Marler LM, Siders JA, Wolters LC, Pettigrew Y, Skitt BL, Allen SD 1992 Comparison of five cultural procedures for isolation of *Clostridium difficile* from stools. J Clin Microbiol 30:514–516. 1537928 10.1128/jcm.30.2.514-516.1992 PMC265091

[9] Aziz RK, Bartels D, Best AA, DeJongh M, Disz T, Edwards RA, Formsma K, Gerdes S, Glass EM, Kubal M, Meyer F, Olsen GJ, Olson R, Osterman AL, Overbeek RA, McNeil LK, Paarmann D, Paczian T, Parrello B, Pusch GD, Reich C, Stevens R, Vassieva O, Vonstein V, Wilke A, Zagnitko O 2008 The RAST server: rapid annotations using subsystems technology. BMC Genomics 9:75. doi:10.1186/1471-2164-9-75. 18261238 PMC2265698

